# State-Driven Adaptive Deep-Unfolded PGA Algorithm for Hybrid Beamforming in MIMO-JCAS Systems

**DOI:** 10.3390/s26103276

**Published:** 2026-05-21

**Authors:** Fulai Liu, Zihao Wang, Yan Gao, Zhuoyi Yao

**Affiliations:** 1 Laboratory of Electromagnetic Environment Cognition and Control Utilization, and Hebei Key Laboratory of Marine Perception Network and Data Processing, Northeastern University at Qinhuangdao, Qinhuangdao 066004, China; 2 School of Computer Science and Communication Engineering, Northeastern University at Qinhuangdao, Qinhuangdao 066004, China; 202312051@stu.neuq.edu.cn; 3 School of Computer Science and Engineering, Northeastern University, Shenyang 110819, China; zhuoyiyao163@163.com

**Keywords:** joint communication and sensing, hybrid beamforming, deep-unfolded network, adaptive hyperparameter control

## Abstract

In massive multiple-input multiple-output (MIMO) joint communication and sensing (JCAS) systems, hybrid beamforming (HBF) has attracted much attention because it can provide a favorable tradeoff between beamforming gain and hardware cost. However, HBF design in MIMO-JCAS systems is highly challenging. The main reasons are the strong coupling between the analog and digital precoders in joint communication-sensing optimization and the high-dimensional search space caused by large-scale antenna arrays. In this paper, a state-driven adaptive deep-unfolded hybrid beamforming algorithm is proposed for MIMO-JCAS systems. Specifically, the analog precoder update is redesigned in a manifold-based form to better match the geometry of the constant-modulus constraint, while the digital precoder update is enhanced by a learnable gradient-balancing mechanism to alleviate the dynamic imbalance between the communication-rate gradient and the sensing-error gradient. Furthermore, a lightweight state-driven control network is introduced to generate scaling factors for the hyperparameters according to the current iteration state, so that the unfolded model can adapt its update behavior during optimization. Different from conventional deep-unfolded methods with static hyperparameters during inference, the proposed method provides a more effective optimization strategy for the dynamic communication-sensing tradeoff in MIMO-JCAS hybrid beamforming. Simulation results demonstrate the effectiveness of the proposed state-driven adaptive deep-unfolded method. Compared with the conventional deep-unfolded projected gradient ascent (PGA) algorithm with 20 inner iterations, the proposed method improves the joint objective, while achieving faster convergence and stronger robustness.

## 1. Introduction

Sixth-generation mobile networks are expected to support many emerging applications, including Internet of Things (IoT) networks, vehicle-to-everything (V2X) services, and unmanned aerial vehicle (UAV) networks. These applications require high-quality wireless connectivity and reliable sensing capability. To meet the growing demand for communication and sensing, multiple-input multiple-output joint communication and sensing (MIMO-JCAS) systems have attracted increasing interest. In MIMO-JCAS systems, the wireless infrastructure is expected to deliver high-rate data streams and provide transmit-side spatial sensing capability, which is commonly characterized by the quality of the generated sensing beam pattern or the accuracy of the approximated transmit covariance [1,2].

However, because the hardware cost and power consumption of fully digital precoding become prohibitive in large-scale antenna arrays, hybrid beamforming (HBF) has been widely studied in MIMO-JCAS systems [3,4]. In HBF architectures, the overall precoder is decomposed into a low-dimensional digital precoder and an analog precoder implemented by phase shifters, thereby achieving an attractive tradeoff between beamforming gain and hardware efficiency. Nevertheless, HBF design for MIMO-JCAS systems remains challenging. A direct difficulty comes from the element-wise constant-modulus constraint imposed by the analog phase-shifter network, which leads to a nonconvex feasible set. It should be noted that such constant-modulus or unit-modulus constraints are not unique to analog precoding, and similar constraints also arise in other wireless scenarios, such as reconfigurable intelligent surface (RIS)-empowered networks, where the reflecting phase shifts are typically subject to unit-modulus constraints. The constraints can be handled by various optimization-based and AI-based methods [5]. Therefore, the main difficulty in the considered MIMO-JCAS HBF problem does not stem from the constant-modulus constraint alone, but from the strong coupling between the digital and analog precoders, and the additional need to coordinate communication-rate maximization and sensing-performance optimization within the same hybrid beamforming design [6].

To address the hybrid beamforming design problem in MIMO-JCAS systems, various model-based optimization approaches have been developed. Among them, alternating optimization (AO)-based methods are widely adopted because the original nonconvex problem can be decomposed into tractable subproblems. For example, in dual-function radar-communication (DFRC) hybrid beamforming design, the optimization problem is decomposed into three subproblems and is solved iteratively by AO, so that a favorable tradeoff between communication performance and radar beampattern matching is achieved [7]. More realistic DFRC hybrid architectures are considered in [8], where fully connected and partially connected structures are studied. For these two architectures, MADMM and RPM-TR are developed, respectively, and good communication rates as well as desirable radar beampattern performance are achieved. The corresponding design is further extended to wideband orthogonal frequency division multiplexing (OFDM)-based DFRC systems in [9], where the transmit and receive hybrid beamformers are jointly optimized in the presence of frequency selectivity. Although AO-based approaches are capable of producing feasible solutions, they are often sensitive to hyperparameters and are likely to converge to poor local optima, which limits their performance in complex scenarios [10,11,12].

To reduce online computational cost and latency, data-driven deep learning (DL) methods are also introduced into JCAS and HBF design. In JCAS systems, autoencoder-based end-to-end frameworks are studied, where waveforms or transceiver mappings for communication and sensing tasks are directly learned [13,14]. In HBF design, data-driven neural architectures are also used to learn mappings from channel-related observations to hybrid precoders, thereby improving real-time capability and reducing explicit optimization effort during inference [15,16]. These studies show that complex nonlinear relationships in wireless systems can be effectively learned by DL. However, purely data-driven approaches usually require large training datasets, and their model interpretability is generally weaker than that of model-based optimization methods. These limitations become more significant when physically meaningful constraints and iterative decision logic need to be preserved.

Deep unfolding is an effective framework for alleviating the limitations of AO-based optimization and purely data-driven DL methods. By transforming an iterative optimization procedure into a neural network with a finite number of layers, deep unfolding preserves algorithmic interpretability while enabling key hyperparameters to be learned from data [17,18,19,20]. In hybrid beamforming design, alternating optimization can be unfolded into a trainable architecture, where the analog precoder is updated through a lightweight unfolded network and the digital precoder is obtained by a closed-form step, so that computational complexity is reduced while good beamforming performance is maintained [21]. Beyond hybrid beamforming, projected-gradient-type deep unfolding has also been investigated in other wireless optimization tasks. A mixture-of-experts-augmented deep unfolding framework is developed for activity detection in IRS-aided massive access systems, where the unfolded projected-gradient updates are used to improve detection robustness under mixed channel fading conditions [22]. More closely related to MIMO-JCAS systems, a modified projected gradient ascent (PGA) procedure can be unfolded, where repeated analog updates and a weighted sensing-gradient term are introduced, so that convergence is improved and favorable communication-sensing performance is achieved [23]. These studies indicate that deep unfolding provides a practical balance among interpretability, convergence efficiency, and online complexity for wireless optimization problems, while its use in MIMO-JCAS HBF design still requires careful treatment of analog-digital precoder coupling, constant-modulus constraints, and communication-sensing tradeoffs.

Despite these advances, an important limitation remains. In most existing deep-unfolded designs, the hyperparameters learned during training remain fixed during inference. As a result, the update strategy cannot adapt to the current optimization state, even though the relative importance of communication-rate improvement and sensing-error reduction may vary substantially across iterations. In addition, the two precoder updates are affected by different structural difficulties. For the analog precoder, Euclidean-space updates followed by projection may not align well with the geometry induced by the constant-modulus constraint, which can reduce update efficiency. For the digital precoder, the relative magnitudes of the communication-rate gradient and the sensing-error gradient may vary dynamically during the iterative process, so a fixed balancing factor may not always provide a desirable update direction. These issues motivate the development of a more adaptive unfolded HBF design for MIMO-JCAS systems.

To address the above issues, this paper proposes a state-driven adaptive deep-unfolded PGA algorithm for hybrid beamforming in MIMO-JCAS systems. The main contributions of this paper are summarized as follows:

- A state-driven adaptive hyperparameter control mechanism is developed for unfolded HBF in MIMO-JCAS systems to better handle the dynamic communication-sensing tradeoff during iterative hybrid precoder optimization. Specifically, a lightweight deep neural network (DNN) is introduced to generate scaling factors for the analog step size, the digital step size, and the gradient-balancing factor according to the current iteration state. The state vector is constructed from the current joint objective, communication rate, sensing error, gradient norms, hyperparameter statistics, iteration index, and objective variation, so that the controller can capture the evolving optimization status of the coupled analog-digital precoder updates. In this way, the unfolded network is enabled to adapt its update behavior to different optimization stages, which improves the efficiency and robustness of hybrid beamforming optimization compared with using static hyperparameters.
- A manifold-based update framework is developed for the analog precoder. Instead of directly performing Euclidean updates followed by projection, the analog-precoder gradient is projected onto the tangent space associated with the complex circle manifold, and the update is then carried out along a direction that is more consistent with the geometry of the unit-modulus constraint set. Specifically, the standard Euclidean gradient is first computed. To prevent the update from violating hardware constraints, this gradient is projected onto the local tangent space of the current precoder state. This crucial step strips away the invalid gradient components that attempt to alter signal amplitudes, isolating only the valid components that modify phases. The analog precoder is then updated along this geometry-aware tangent direction. Finally, an element-wise normalization is applied as a retraction step to precisely pull the intermediate state back onto the feasible circular boundary. As a result, the analog-precoder update becomes better matched to the feasible set, which improves the update efficiency of shallow unfolded models.
- A learnable and adaptive gradient-balancing strategy is incorporated into the digital precoder update. To address the dynamic imbalance between the communication-rate gradient and the sensing-error gradient during joint optimization, the fixed balancing factor used in conventional methods is extended to a trainable parameter and is further adjusted by the state-driven controller. This design allows the digital precoder to be updated with a direction that is better suited to the current rate–sensing tradeoff, thereby improving joint optimization effectiveness.

The remainder of this paper is organized as follows. Section 2 introduces the system and signal model and formulates the hybrid beamforming optimization problem. Section 3 presents the deep-unfolded PGA algorithm based on Riemannian manifold optimization and discusses the gradient imbalance between the communication and sensing terms in the digital precoder update to introduce the learnable gradient balancing factor. Based on Section 3, a state-driven adaptive mechanism and the corresponding adaptive deep-unfolded PGA model are developed in Section 4. Section 5 reports the numerical results and provides the corresponding analysis. Finally, Section 4 concludes the paper.

Throughout this paper, bold uppercase letters denote matrices, bold lowercase letters denote vectors, and scalar quantities are represented by regular letters. The operators ${\left(\cdot \right)}^{T}$, ${\left(\cdot \right)}^{*}$, and ${\left(\cdot \right)}^{H}$ denote the transpose, complex conjugate, and Hermitian transpose, respectively. The sets of *n*-dimensional complex-valued (real-valued) vectors and N×N complex-valued (real-valued) matrices are denoted by $C^{n}$ ($R^{n}$) and $C^{N\times N}$ ($R^{N\times N}$), respectively. The symbols ⊗ and ⊙ denote the Kronecker product and the Hadamard product, respectively. The notations |·|, ${∥\cdot ∥}_{2}$, and ${∥\cdot ∥}_{F}$ represent the modulus of a scalar, the Euclidean norm, and the Frobenius norm, respectively. In addition, vec(·) and tr(·) denote vectorization and the trace operation, respectively. The notation $\mathrm{CN}(\mu ,\sigma^{2})$ denotes a complex Gaussian distribution with mean μ and variance $\sigma^{2}$, while U[a,b] denotes the uniform distribution over the interval [a,b]. The notation Re(·) denotes the real part of a complex-valued quantity.

**Figure 1:**
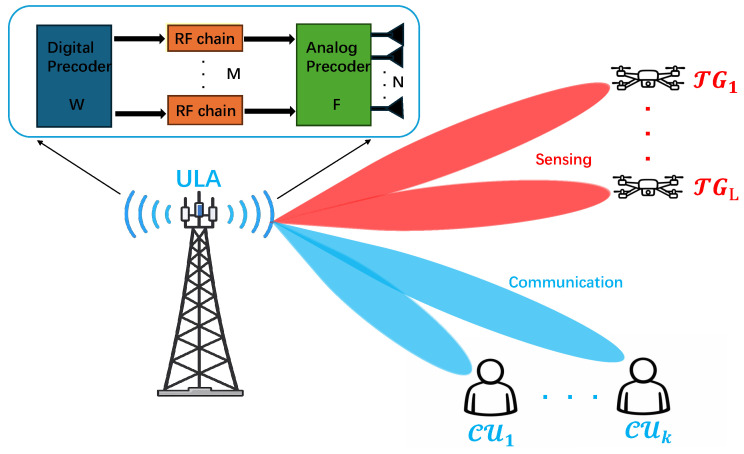
System architecture of the considered MIMO JCAS hybrid beamforming system.

Figure 1 illustrates the system architecture of the considered MIMO-JCAS hybrid beamforming system.

## 2. Signal Model and Problem Formulation

### 2.1. Signal Model

This paper considers a MIMO JCAS system, where the base station (BS) equipped with *N* antennas employs a hybrid precoding architecture to simultaneously transmit communication signals to *K* users and detecting radar signals toward *L* targets. *M* represents the number of RF chains at the BS. Let $s_{k}$ denote the transmitted signal intended for the *k*-th user, and let $w_{k}$ denote its corresponding digital precoding vector. The digital precoding matrix is then given by $W=\left[w_{1},w_{2},\ldots ,w_{K}\right]\in C^{M\times K}$, while the analog precoding matrix is denoted by $F\in C^{N\times M}$, whose entries satisfy the element-wise unit-modulus constraint ${|\left[F\right]}_{n,m}|=1,\;\forall n,m$. In addition, the analog and digital precoders jointly satisfy the transmit power constraint ${∥\mathrm{FW}∥}_{F}^{2}=P_{BS}$, where $P_{BS}$ denotes the transmit power of the base station. For channel modeling, the extended Saleh–Valenzuela channel model is adopted in this paper:

(1) $$h_{k}=\sum_{q=1}^{Q}\alpha_{q,k}\;a\left(\phi_{q,k}\right),$$

where $h_{k}$ denotes the channel from the base station to user *k*, *Q* is the number of propagation paths, and $\alpha_{q,k}$ and $\phi_{q,k}$ represent the complex path gain and the angle of departure (AoD) of the *q*-th path from the base station to user *k*, respectively. Here, $a(\phi_{q,k})$ denotes the transmit array response vector. Assuming a uniform linear array (ULA), the transmit array response vector can be written as:

(2) $$a\left(\phi_{q,k}\right)=\frac{1}{\sqrt{N}}{\left[1,e^{j\pi \sin(\phi_{q,k})},\ldots ,e^{j\left(N-1\right)\pi \sin\left(\phi_{q,k}\right)}\right]}^{T}.$$

Based on the above definitions, the received signal at user *k* can be expressed as:

(3) $$y_{k}=h_{k}^{H}Fw_{k}s_{k}+h_{k}^{H}\sum_{k^{\prime }\neq k}^{K}Fw_{k^{\prime }}s_{k^{\prime }}+n_{k},$$

where the first term is the desired signal, the second term represents the multiuser interference, and $n_{k}∼\mathrm{CN}\left(0,\sigma_{n}^{2}\right)$ denotes the additive white Gaussian noise at user *k*.

### 2.2. Problem Formulation

In this paper, the sum rate is adopted as the communication performance metric. According to Equation (3), the achievable sum rate of the *K* users can be written as:

(4) $$R=\sum_{k=1}^{K}\log_{2}\left(1+\frac{{\left|h_{k}^{H}Fw_{k}\right|}^{2}}{\sum_{k^{\prime }\neq k}^{K}{\left|h_{k}^{H}Fw_{k^{\prime }}\right|}^{2}+\sigma_{n}^{2}}\right).$$

For sensing performance, the radar probing capability is characterized by the similarity between the transmit beam pattern and the desired beam pattern. The reference transmit covariance matrix is obtained by solving the following convex radar beamforming problem using the MATrix LABoratory (MATLAB R2023b) software for disciplined convex programming (CVX 2.2) toolbox [24,25]:

(5a) $$\begin{array}{cc} \min_{\alpha ,\Psi }\; & \sum_{t=1}^{T}{\left|\alpha P_{d}\left(\theta_{t}\right)-\bar{a}{\left(\theta_{t}\right)}^{H}\Psi \bar{a}\left(\theta_{t}\right)\right|}^{2} \end{array}$$

(5b) $$\begin{array}{cc} \mathrm{subject}\;\mathrm{to}\; & {\left[\Psi \right]}_{n,n}=\frac{P_{BS}}{N},\;\forall n \end{array}$$

(5c) $$\begin{array}{cc} \; & \Psi ⪰0,\;\Psi =\Psi^{H}. \end{array}$$

Here, ${\left\{\theta_{t}\right\}}_{t=1}^{T}$ denotes *T* angular samples defined over the range $\left[-90^{\circ },90^{\circ }\right]$ with an angular resolution of $0.1^{\circ }$; α is a scaling factor; $P_{d}\left(\theta_{t}\right)$ denotes the desired beam pattern; and $a(\theta_{t})$ is the transmit array steering vector corresponding to angle $\theta_{t}$. This paper focuses on transmit beam pattern design for radar sensing, rather than subsequent target detection and localization.

In this paper, the mismatch between the covariance matrix of the transmitted signal and the reference waveform matrix is adopted as the radar performance:

(6) $$\tau ≜{∥FWW^{H}F^{H}-\Psi ∥}_{F}^{2}.$$

By jointly taking the communication and sensing performances into account, the resulting optimization problem can be formulated as:

(7a) $$\begin{array}{cc} \max_{F,W}\; & R-w\tau \end{array}$$

(7b) $$\begin{array}{cc} \mathrm{subject}\;\mathrm{to}\; & {|\left[F\right]}_{n,m}|=1,\;\forall n,m \end{array}$$

(7c) $$\begin{array}{cc} \; & {∥FW∥}_{F}^{2}=P_{BS}. \end{array}$$

Here, *w* is a tradeoff parameter that balances the communication rate and the sensing error. The effect of *w* on the overall model performance will be discussed later in the paper. The weighted formulation in (7) is adopted to balance the communication and sensing objectives in a adjustable way. The objective of this work is to develop a state-driven adaptive unfolded HBF framework that can operate under different communication-sensing tradeoff preferences. The weight factor *w* allows the same unfolded architecture to continuously adjust the tradeoff between rate maximization and sensing-error minimization, without requiring a threshold-dependent algorithm design. Therefore, (7) is used for adjustable communication-sensing tradeoff design, while constrained formulations are more suitable when strict sensing guarantees are required.

## 3. Proposed Design

This section separately discusses the analog and digital precoder updates. For the analog precoder, the difficulty is that the Euclidean update does not naturally satisfy the constant-modulus constraints in Equation (7b). For the digital precoder, the challenge is that the contributions of sensing and communication to the digital update direction are dynamically imbalanced. We consider enhancing the deep-unfolded projected gradient ascent (PGA) framework in two aspects. More specifically, the analog precoder update is extended from the Euclidean space to the manifold space so that the update becomes more consistent with the constraint geometry and more effective in each inner iteration. Meanwhile, the gradient balancing factor $\eta_{\left(i\right)}$ is set as a learnable parameter to balance the sensing and communication gradients, thereby providing a more suitable digital update direction and better optimization efficiency.

### 3.1. Proposed Manifold-Based Analog Update

Following the modified deep-unfolded PGA framework in [23], this paper adopts a nested update schedule to solve the formulated problem, where the step sizes are treated as learnable parameters. Specifically, to alleviate the gradient-scale imbalance between the digital precoder *W* and the analog precoder *F*, the analog precoder *F* is refined for *J* PGA-based steps before each update of the digital precoder *W*. Therefore, the terms “inner loop” and “outer loop” refer to the update schedule of the modified PGA procedure, rather than to a conventional AO hierarchy between *W* and *F*.

To address the geometric mismatch between the original Euclidean-space update and the constant-modulus constraint, we develop a manifold-based update for the analog precoder within the deep-unfolded PGA framework. Instead of applying a Euclidean update followed by direct projection, the Euclidean gradient of the objective function with respect to the analog precoder is first calculated, denoted by $G_{\left(i,j\right)}$. Then, it is projected onto the tangent space to obtain the Riemannian gradient $G_{R(i,j)}$, based on which the analog precoder is updated along a geometry-aware direction. This redesign makes the update of the analog precoder more consistent with the underlying constraint geometry and improves the effectiveness of each inner iteration. Let $\mu_{\left(i,j\right)}$ denote the update step size of the analog precoder in the *j*-th inner iteration of the *i*-th outer layer. The analog update process is as follows:

(8) $$G_{\left(i,j\right)}=\nabla_{F^{*}}R-w\nabla_{F^{*}}\tau$$

(9) GR(i,j)=G(i,j)−ReG(i,j)⊙F(i,j)*⊙F(i,j),

Here, Equation (9) provides a geometric projection of the Euclidean gradient onto the tangent space of the complex circle manifold. Physically, the analog precoder $F_{\left(i,j\right)}$ is implemented by RF phase shifters, whose amplitudes are fixed by the constant-modulus constraint and whose phases are adjustable. The raw Euclidean gradient $G_{\left(i,j\right)}$ generally contains both tangential components, which correspond to feasible first-order phase variations, and normal components, which correspond to infeasible first-order amplitude variations.

Geometrically, the term ReG(i,j)⊙F(i,j)*⊙F(i,j) represents the normal component of the gradient along the radial direction of the complex circle. This component tends to drive the analog precoder away from the constant-modulus manifold and is therefore inconsistent with the RF phase-shifter implementation. By subtracting this normal component from the Euclidean gradient, Equation (9) extracts the tangent-space direction $G_{R(i,j)}$, which is consistent with the local geometry of the unit-modulus constraint. Therefore, the projected gradient provides a physically meaningful phase-update direction for the analog precoder.

(10) $${\tilde{F}}_{\left(i,j+1\right)}=F_{\left(i,j\right)}+\mu_{\left(i,j\right)}G_{R(i,j)},$$

(11) $${\left[F_{\left(i,j+1\right)}\right]}_{nm}=\frac{{\left[{\tilde{F}}_{\left(i,j+1\right)}\right]}_{nm}}{\left|{\left[{\tilde{F}}_{\left(i,j+1\right)}\right]}_{nm}\right|},\;\forall n,m.$$

In contrast to the analog precoder F, the digital precoder W is not subject to the element-wise constant-modulus constraint. Therefore, the complex circle manifold used for the analog precoder F is not directly applicable to the digital precoder W. In this paper, the manifold-based update is retained for the analog precoder F throughout all inner iterations, while the digital precoder W is updated in the Euclidean space and then normalized to satisfy the transmit-power constraint. Let $\lambda_{\left(i\right)}$ and $\eta_{\left(i\right)}$ denote the digital update step size and the gradient-balancing factor at the *i*-th outer iteration, respectively. Accordingly, the digital update is given by

(12) $$W_{\left(i+1\right)}=W_{\left(i\right)}+\lambda_{\left(i\right)}\left(\nabla_{W^{*}}R-\eta_{\left(i\right)}w\nabla_{W^{*}}\tau \right){|}_{W=W_{\left(i\right)}},$$

(13) $$W_{\left(i+1\right)}=\frac{P_{BS}W_{\left(i+1\right)}}{{∥F_{\left(i+1\right)}W_{\left(i+1\right)}∥}_{F}}.$$

The gradients of the communication rate and the sensing error with respect to the digital and analog precoding matrices are given in [23]:

(14) $$\begin{array}{cc} \nabla_{F^{*}}R= & \sum_{k=1}^{K}\frac{{\tilde{H}}_{k}FV}{\ln2\left(\mathrm{tr}\left(FVF^{H}{\tilde{H}}_{k}\right)+\sigma_{n}^{2}\right)}\\ \; & -\sum_{k=1}^{K}\frac{{\tilde{H}}_{k}FV_{\bar{k}}}{\ln2\left(\mathrm{tr}\left(FV_{\bar{k}}F^{H}{\tilde{H}}_{k}\right)+\sigma_{n}^{2}\right)}, \end{array}$$

(15) $$\begin{array}{cc} \nabla_{W^{*}}R= & \sum_{k=1}^{K}\frac{{\bar{H}}_{k}W}{\ln2\left(\mathrm{tr}\left(WW^{H}{\bar{H}}_{k}\right)+\sigma_{n}^{2}\right)}\\ \; & -\sum_{k=1}^{K}\frac{{\bar{H}}_{k}W_{\bar{k}}}{\ln2\left(\mathrm{tr}\left(W_{\bar{k}}W_{\bar{k}}^{H}{\bar{H}}_{k}\right)+\sigma_{n}^{2}\right)}, \end{array}$$

(16) $$\nabla_{F^{*}}\tau =2\left(FWW^{H}F^{H}-\Psi \right)FWW^{H},$$

(17) $$\nabla_{W^{*}}\tau =2F^{H}\left(FWW^{H}F^{H}-\Psi \right)FW,$$

where the relevant matrices are defined as:

(18) $$V≜WW^{H}\in C^{M\times M},\;V_{k}≜W_{k}W_{k}^{H}\in C^{M\times M},$$

(19) $${\bar{H}}_{k}≜h_{k}h_{k}^{H}\in C^{N\times N},\;{\tilde{H}}_{k}≜F^{H}{\bar{H}}_{k}F\in C^{M\times M}.$$

Here, $W_{\bar{k}}\in C^{M\times K}$ is obtained by setting the *k*-th column of W to zero.

### 3.2. Learnable Gradient Balancing

This paper will describe how much the performance has improved with the manifold-based update mechanism through the simulation results. However, only improving the update space is still insufficient to fully exploit the model’s capability. That is because the update direction is also influenced by the balance between $\nabla_{W^{*}}R$ and $\nabla_{W^{*}}\tau$. More specifically, the update direction for the digital precoder can be written as:

(20) $$\nabla_{W^{*}}R-\eta_{\left(i\right)}w\nabla_{W^{*}}\tau .$$

Solving the numerical imbalance between $\nabla_{W^{*}}R$ and $\nabla_{W^{*}}\tau$ is important to the effectiveness of the joint update. The gradient balancing factor $\eta_{\left(i\right)}$ is introduced to handle this problem. However, the conventional deep-unfolded PGA algorithm sets $\eta_{\left(i\right)}$ as a fixed parameter, which ignores the fact that the relative contributions of the communication and sensing gradients vary across iterations.

**Figure 2:**
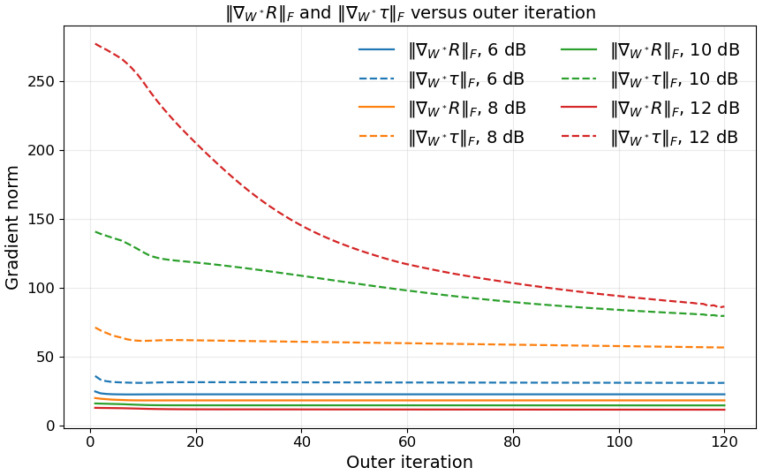
Evolution of $∥\nabla_{W^{*}}{R∥}_{F}$ and $∥\nabla_{W^{*}}{\tau ∥}_{F}$ under different SNR conditions.

Figure 2 shows the evolution of $\nabla_{W^{*}}R$ and $\nabla_{W^{*}}\tau$ during the iterative process under different signal-to-noise ratio (SNR) conditions. Here, the superscript * denotes complex conjugation, and $\nabla_{W^{*}}$ denotes the Wirtinger gradient with respect to the complex conjugate variable $W^{*}$. Different colors are used to distinguish different SNR values, while solid and dashed lines are used to distinguish $∥\nabla_{W^{*}}{R∥}_{F}$ and $∥\nabla_{W^{*}}{\tau ∥}_{F}$, respectively. As the iterations proceed, $\nabla_{W^{*}}R$ remains within a relatively stable range throughout the process. By contrast, under high-SNR conditions, $\nabla_{W^{*}}\tau$ decreases significantly with the iteration index. That is, the balance between the communication gradient and the sensing gradient is dynamic and stage-dependent. When one gradient term dominates the other in magnitude, the combined update direction becomes biased toward only one objective, making the rate-sensing tradeoff harder to control across iterations. To address this issue, we treat $\eta_{\left(i\right)}$ as a learnable parameter, allowing the weight of $\nabla_{W^{*}}\tau$ to be adjusted automatically during training.

In summary, manifold optimization mainly resolves the issue of geometric consistency between the update of the analog precoder and the unit-modulus constraint, whereas the learnable gradient balancing factor $\eta_{\left(i\right)}$ mainly guides a better update direction for the digital precoder. They improve the overall performance of the unfolded network under a limited number of layers together.

## 4. State-Driven Adaptive Deep-Unfolded Network

We improve the overall network performance through the learnable $\eta_{\left(i\right)}$; however, the learned $\eta_{\left(i\right)}$ is still static and associated with the layer index, which leads to poor model robustness. The step-size parameters are in a similar situation. This motivates us to introduce an iteration-state-driven hyperparameter control mechanism to adjust the parameters according to the current iteration state, thereby enhancing the optimization efficiency, model robustness, and final joint performance of shallow unfolded networks.

### 4.1. Definition of the State Vector

To enable dynamic adjustment of the step sizes $\mu_{\left(i,j\right)}$ and $\lambda_{\left(i\right)}$ and the gradient balancing factor $\eta_{\left(i\right)}$ while preserving the interpretability of deep unfolding, we define a lightweight control neural network. Its role is to take the current iteration-state vector as input and output the scaling factors for the step sizes and the gradient balancing factor. The state vector is defined to characterize the state in the iteration, which can be written as:

(21) $$s_{i}=\left[\begin{array}{c} J_{\left(i\right)}\\ R_{\left(i\right)}\\ \tau_{\left(i\right)}\\ ∥\nabla_{F^{*}}R_{\left(i\right)}{∥}_{F}\\ ∥\nabla_{F^{*}}\tau_{\left(i\right)}{∥}_{F}\\ ∥\nabla_{W^{*}}R_{\left(i\right)}{∥}_{F}\\ ∥\nabla_{W^{*}}\tau_{\left(i\right)}{∥}_{F}\\ {\bar{\mu }}_{\left(i\right)}\\ \lambda_{\left(i\right)}\\ \eta_{\left(i\right)}\\ i/\left(I-1\right)\\ \Delta J_{\left(i\right)} \end{array}\right].$$

Here, $J_{\left(i\right)}$, $R_{\left(i\right)}$, and $\tau_{\left(i\right)}$ denote the joint objective, the communication rate, and the sensing error at the *i*-th outer iteration. These features provide a direct evaluation of the current optimization stage and explicitly quantify the real-time tradeoff status between the communication rate and the sensing error. Moreover, $∥\nabla_{F^{*}}R_{\left(i\right)}{∥}_{F}$ and $∥\nabla_{F^{*}}\tau_{\left(i\right)}{∥}_{F}$ denote the Frobenius norms of the gradients of the communication rate and the sensing error with respect to the analog precoder, respectively, while $∥\nabla_{W^{*}}R_{\left(i\right)}{∥}_{F}$ and $∥\nabla_{W^{*}}\tau_{\left(i\right)}{∥}_{F}$ denote the corresponding Frobenius norms with respect to the digital precoder. The gradient norms serve as the fundamental driving forces of the updates. Including them is crucial because they allow the controller to perceive which objective is dominating the descent direction at any given moment, providing necessary numerical foundations for adaptively generating the gradient balancing factor $\eta_{\left(i\right)}$. $\lambda_{\left(i\right)}$ and $\eta_{\left(i\right)}$ denote the step size and gradient balancing factor of the *i*-th outer iteration. We further introduce the statistic ${\bar{\mu }}_{\left(i\right)}$ to characterize the overall hyperparameter level in the current layer, which is defined as:

(22) $${\bar{\mu }}_{\left(i\right)}=\frac{1}{J}\sum_{j=1}^{J}\mu_{\left(i,j\right)},$$

where ${\bar{\mu }}_{\left(i\right)}$ is the average size of the *i*-th inner step.

By feeding the current base scale of the hyperparameters (${\bar{\mu }}_{\left(i\right)}$, $\lambda_{\left(i\right)}$, $\eta_{\left(i\right)}$) into the controller, the network gains awareness of its own parameter space. This self-awareness is essential to strictly prevent the generated scaling factors from causing step-size explosion or vanishing gradients.

In addition, the normalized index $\frac{i}{I-1}$ is introduced to characterize the depth of the current iteration layer, which enables the network to distinguish between early-stage exploration and late-stage exploitation.

To capture the changing trend of the joint objective, $\Delta J_{\left(i\right)}$ is introduced to describe the improvement achieved during optimization and to provide additional dynamic information, which is defined as:

(23) $$\Delta J_{\left(i\right)}=\left\{\begin{array}{cc} 0, & i=0,\\ J_{\left(i\right)}-J_{\left(i-1\right)}, & i>0, \end{array}\right.$$

where $\Delta J_{\left(i\right)}$ denotes the change in the joint objective at the *i*-th outer iteration, which informs the controller whether the optimization is improving steadily or plateauing, allowing it to adaptively decay or adjust the step sizes to guarantee stable convergence.

### 4.2. Adaptive Network Outputs and Update Procedure

In summary, the constructed state vector includes the joint objective, the communication rate, the sensing error, the gradient information of the communication and sensing terms with respect to the precoders, the hyperparameter statistics, the normalized layer index, and the variation trend of the joint objective. These features provide a comprehensive description of the optimization state during iteration. Based on the state vector, the lightweight control neural network outputs the scaling factors to dynamically adjust the hyperparameters at the current iteration.

The hyperparameter scaling factors are denoted by $\alpha_{\mu }^{\left(i\right)}$, $\alpha_{\lambda }^{\left(i\right)}$, and $\alpha_{\eta }^{\left(i\right)}$, corresponding to the scaling of the inner-loop step size, the outer-loop step size, and the gradient balancing factor at the *i*-th outer iteration, respectively. With these definitions, the update procedure is rewritten as:

(24a) $$\begin{array}{cc} \mu_{\left(i,j\right)}^{\mathrm{eff}} & =\mu_{\left(i,j\right)}\alpha_{\mu }^{\left(i\right)}, \end{array}$$

(24b) $$\begin{array}{cc} \lambda_{\left(i\right)}^{\mathrm{eff}} & =\lambda_{\left(i\right)}\alpha_{\lambda }^{\left(i\right)}, \end{array}$$

(24c) $$\begin{array}{cc} \eta_{\left(i\right)}^{\mathrm{eff}} & =\eta_{\left(i\right)}\alpha_{\eta }^{\left(i\right)}. \end{array}$$

The analog precoder is then updated according to Equations (8) and (9), and:

(25) $${\tilde{F}}_{\left(i,j+1\right)}=F_{\left(i,j\right)}+\mu_{\left(i,j\right)}^{\mathrm{eff}}G_{R(i,j)},$$

followed by Equation (11).

The digital precoder is updated as:

(26) $$W_{\left(i+1\right)}=W_{\left(i\right)}+\lambda_{\left(i\right)}^{\mathrm{eff}}\left(\nabla_{W^{*}}R-\eta_{\left(i\right)}^{\mathrm{eff}}\;w\;\nabla_{W^{*}}\tau \right){|}_{W=W_{\left(i\right)}},$$

followed by the normalization operation in Equation (13).

### 4.3. State-Driven Adaptive Deep-Unfolded Network Architecture

Consider an *I*-layer deep-unfolded network based on the modified PGA procedure, which aims to output a feasible hybrid precoder with improved communication and sensing performance by maximizing the joint objective R−wτ. The alternating iterative procedure of the original PGA algorithm is mapped into a deep-unfolded network architecture, where the outer loop and inner loop correspond to the updates of the digital precoder W and the analog precoder F, respectively. Building on this framework, a manifold-based update mechanism is introduced into the inner loop to better accommodate the unit-modulus constraint, while a learnable gradient balancing factor is incorporated into the outer loop. Based on the current iteration state, a lightweight control neural network generates the scaling factors. Specifically, the network output is passed through a sigmoid function and then linearly mapped to predefined positive intervals, so that the hyperparameters can remain bounded and numerically stable during inference. The overall procedure is summarized in Algorithm 1.

**Figure 3:**
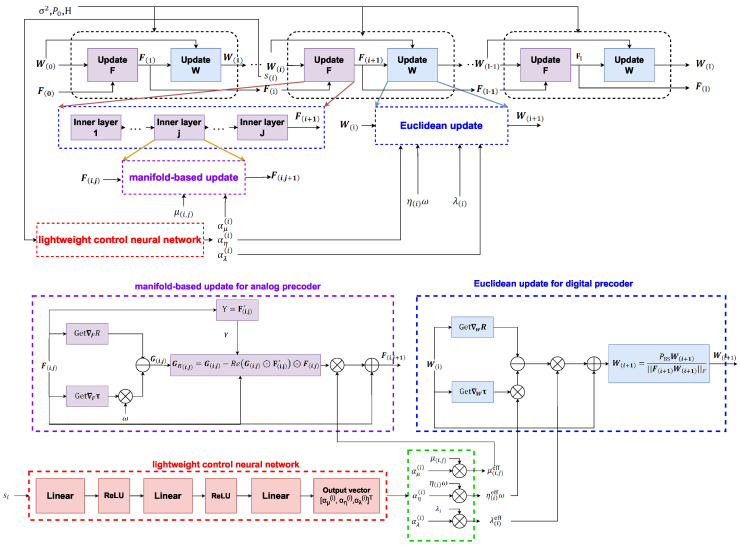
Illustration of the proposed model.

Figure 3 illustrates the architecture of the proposed model, including the manifold-based analog update, the Euclidean digital update, and the lightweight control neural network. Different colored arrows and boxes are used to distinguish different update flows and functional modules in the unfolded architecture.
**Algorithm 1** State-Driven Adaptive Deep-Unfolded projected gradient ascent**Require:** 
H, $P_{\mathrm{BS}}$, *w*, Ψ, the trained base hyperparameters {μ,λ,η}, and the trained control network.**Ensure:** 
F and W.1:  **Initialization:** Generate $\left\{F_{\left(0,0\right)},W_{\left(0\right)}\right\}$, and set $J_{\mathrm{prev}}=0$.2:  **for** i=0 to I−1 **do**3:      Obtain $R_{\left(i\right)}$, $\tau_{\left(i\right)}$, and $J_{\left(i\right)}=R_{\left(i\right)}-w\tau_{\left(i\right)}$.4:      Obtain ${\bar{\mu }}_{\left(i\right)}$ from Equation (22), and obtain $\Delta J_{\left(i\right)}$ from Equation (23).5:      Form the state vector $s_{i}$ based on Equation (21).6:      Generate $\alpha_{\mu }^{\left(i\right)}$, $\alpha_{\lambda }^{\left(i\right)}$, and $\alpha_{\eta }^{\left(i\right)}$ from $s_{i}$ via the lightweight control network.7:      Obtain $\lambda_{\left(i\right)}^{\mathrm{eff}}$ from Equation (24b), and obtain $\eta_{\left(i\right)}^{\mathrm{eff}}$ from Equation (24c).8:      Set $F_{\left(i,0\right)}=F_{\left(i\right)}$.9:      **for** j=0 to J−1 **do**10:          Obtain $\mu_{\left(i,j\right)}^{\mathrm{eff}}$ from Equation (24a).11:          Obtain the gradients $\nabla_{F^{*}}R$ and $\nabla_{F^{*}}\tau$ at         $\left(F,W\right)=\left(F_{\left(i,j\right)},W_{\left(i\right)}\right)$ based on Equations (14) and (16).12:          Obtain $G_{\left(i,j\right)}$ from Equation (8), and obtain $G_{R(i,j)}$ from Equation (9).13:          Obtain $F_{\left(i,j+1\right)}$ based on Equations (25) and (11).14:    **end for**15:    Set $F_{\left(i+1\right)}=F_{\left(i,J\right)}$.16:    Obtain the gradients $\nabla_{W^{*}}R$ and $\nabla_{W^{*}}\tau$ at         $\left(F,W\right)=\left(F_{\left(i+1\right)},W_{\left(i\right)}\right)$ based on Equations (15) and (17).17:    Obtain $W_{\left(i+1\right)}$ based on Equation (26) using $\lambda_{\left(i\right)}^{\mathrm{eff}}$ and $\eta_{\left(i\right)}^{\mathrm{eff}}$.18:    Apply the normalization in Equation (13).19:    Set $J_{\mathrm{prev}}=J_{\left(i\right)}$.20:**end for**21:**return** $F_{\left(I\right)}$ and $W_{\left(I\right)}$ as the solution to F and W.

### 4.4. Model Training

Based on the above architecture, the loss function is defined as:

(27) L=−(R−wτ).

The proposed network is trained in an end-to-end unsupervised manner using PyTorch (version 2.5.1). The simulation setup is specified as L=3, K=M=4, and N=64, with $\sigma_{n}^{2}=1$ and w=0.3. To allow a fair comparison among different methods on a unified dataset, the same channel-data setting as in [23] is adopted. The unfolded architecture uses I=120 outer iterations and J=10 inner iterations. The learnable base step sizes are initialized to $10^{-2}$. The gradient-balancing parameter is initialized as $\eta_{\left(0\right)}=\frac{1}{N}$. In implementation, this is realized by setting $\eta_{\left(0\right)}=1$ and absorbing the factor $\frac{1}{N}$ into the radar-gradient weight. The state-driven controller is implemented as a two-hidden-layer MLP with dimensions 12–32–32–3, where ReLU is used in the hidden layers and the outputs are mapped to [0.15,3.0]. Adam is adopted for optimization, where the learning rates for the base step sizes, the gradient-balancing parameter, and the controller are set to $10^{-3}$, $10^{-1}$, and $2\times 10^{-3}$, respectively.

## 5. Simulation Results

### 5.1. Computational Complexity Analysis

The computational complexity of the proposed method is mainly determined by the gradient evaluations in the analog and digital precoder updates. Let $C_{F}$ and $C_{W}$ denote the complexity of one analog and one digital precoder update, respectively. For the analog precoder update, the computation of $\nabla_{F^{*}}R$ has complexity $O(NM^{2}K)$, while the computation of $\nabla_{F^{*}}\tau$ involves the covariance mismatch term $FWW^{H}F^{H}-\Psi$ and has dominant complexity $O(N^{2}K)$. The manifold projection and retraction are element-wise operations with complexity O(NM), which is negligible compared with the gradient computations. Therefore, the complexity of one analog precoder update is given by

(28) $$C_{F}=O\left(\max\left(NM^{2}K,N^{2}K\right)\right).$$

For the digital precoder update, the computation of $\nabla_{W^{*}}R$ has complexity $O(KNM+M^{2}K^{2})$, while the computation of $\nabla_{W^{*}}\tau$ is dominated by the covariance-related matrix operations with complexity $O(N^{2}K)$. The transmit-power normalization requires O(NMK) operations and is not dominant. Therefore, the complexity of one digital precoder update is

(29) $$C_{W}=O\left(\max\left(M^{2}K^{2},N^{2}K\right)\right).$$

Since the analog precoder is updated *J* times and the digital precoder is updated once in each unfolded outer layer, the overall computational complexity before approximation is

(30) $$\begin{array}{cc} C_{\mathrm{total}}=O( & IJ\max\left(NM^{2}K,N^{2}K\right)\\ & +I\max\left(M^{2}K^{2},N^{2}K\right)+IJNM+IC_{\mathrm{MLP}}), \end{array}$$

where IJNM accounts for the additional manifold projection and retraction operations, and $C_{\mathrm{MLP}}$ denotes the forward-pass complexity of the lightweight state-driven controller.

For typical HBF transceivers, it is generally true that the number of transmit antennas is much larger than the number of RF chains and users, i.e., N≫M,K [23].

Under this commonly used HBF setting, the covariance-related terms dominate the complexity, and the above expression can be approximated as

(31) $$C_{\mathrm{total}}=O\left(IJN^{2}K\right).$$

Therefore, compared with the conventional unfolded PGA algorithm, the proposed method preserves the same dominant complexity order. The additional manifold projection/retraction and state-driven controller introduce only O(IJNM) and $O(IC_{\mathrm{MLP}})$ overheads, respectively, which are negligible compared with the dominant gradient computations.

### 5.2. Communication and Sensing Performance

We next evaluate the performance of the proposed improved deep-unfolded PGA algorithm in terms of communication and sensing performance. For comparison, the following methods are selected as baseline algorithms.

- Conventional deep-unfolded PGA algorithm: Two settings with *J* = 10 and *J* = 20 inner iterations are considered to compare the performance of the proposed method [23].
- MADMM-RCG algorithm: To preserve the structural characteristics of the original method as much as possible, the migrated implementation still adopts auxiliary-variable splitting, ADMM-based alternating updates, and the Polak–Ribière conjugate gradient (Polak–RCG) method with Armijo line search on the analog side under the complex circle manifold framework. Meanwhile, the optimization objective is replaced with R−wτ [8].
- Fully digital ZF beamforming: This method considers only communication performance and is adopted as a communication-oriented reference baseline without the hybrid analog–digital hardware constraint. It is used only for comparison and is not regarded as an achievable-rate upper bound.

In experiments, the tradeoff parameter is set to w=0.3, and the signal-to-noise ratio is set to SNR=12dB. The corresponding numerical results are shown in the figures.

For clarity, the abbreviations used in the legends of the following performance figures are defined as follows.

- **UPGA-J10** denotes the conventional unfolded projected gradient ascent algorithm with J=10 inner iterations [23].
- **UPGA-J20** denotes the conventional unfolded projected gradient ascent algorithm with J=20 inner iterations [23].
- **UPGA-J10-eta** denotes the unfolded PGA algorithm with J=10 inner iterations and the proposed learnable gradient-balancing factor, but without the manifold-based analog update and without the state-driven controller.
- **UPGA-J10-Manifold** denotes the unfolded PGA algorithm with J=10 inner iterations and the proposed manifold-based analog update, but without the learnable gradient-balancing factor and without the state-driven controller.
- **UPGA-J10-Adaptive** denotes the complete proposed method, which includes the manifold-based analog update, the learnable gradient-balancing factor, and the state-driven adaptive controller.
- **MADMM-RCG** denotes the MADMM-RCG baseline algorithm adapted from [8].
- **Fully digital ZF** denotes the fully digital zero-forcing beamforming baseline without the hybrid analog–digital hardware constraint.

**Figure 4:**
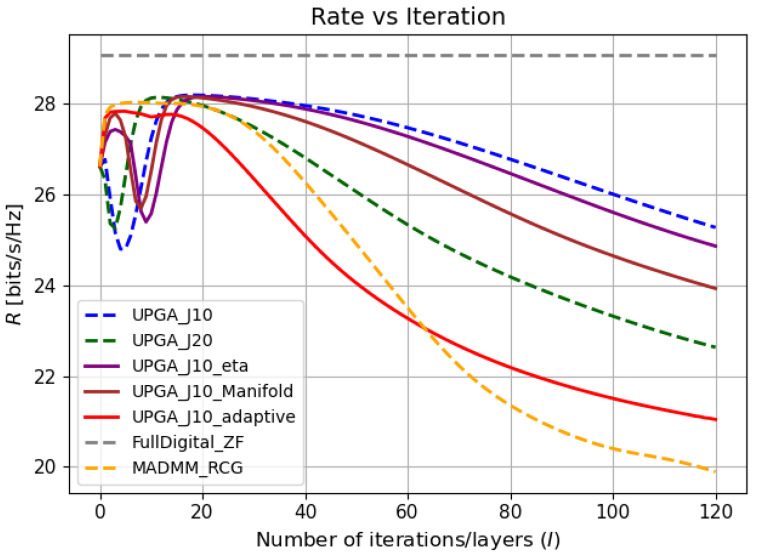
Rate versus iteration.

**Figure 5:**
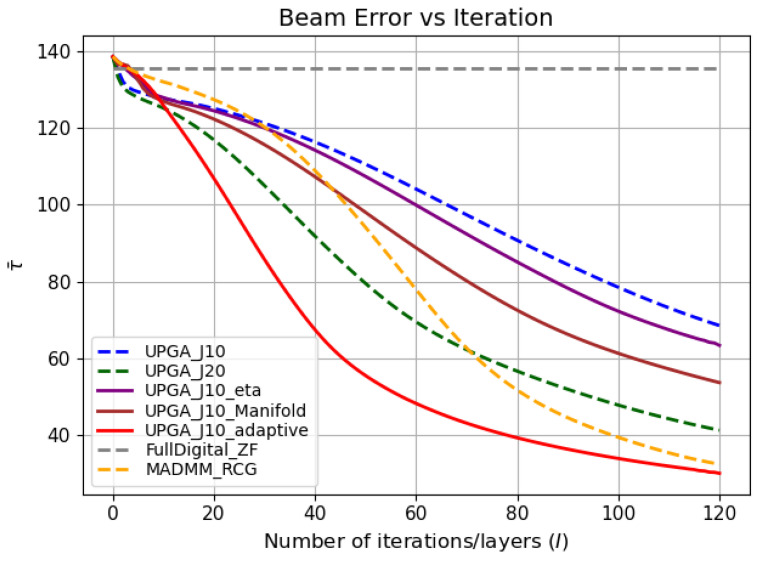
Beam error versus iteration.

**Figure 6:**
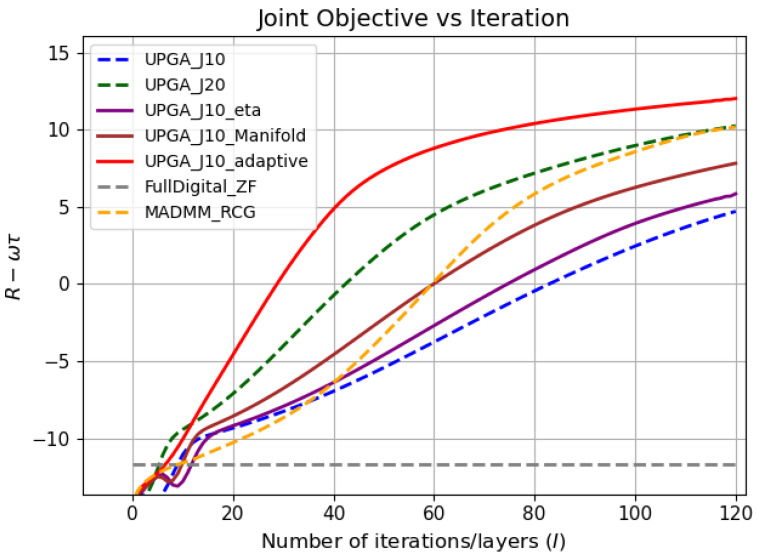
Joint objective versus iteration.

Figure 4, Figure 5 and Figure 6 jointly illustrate the iterative evolution of the compared methods in terms of communication rate, sensing error, and the joint objective R−wτ, respectively. These three metrics should be interpreted together. This is because the proposed method targets joint communication–sensing optimization rather than communication-rate maximization alone. It can be observed that the proposed state-driven adaptive deep-unfolded model maintains a competitive communication rate while achieving the lowest sensing error during the iterative process. As a result, it obtains the highest joint objective, which indicates a more favorable communication–sensing tradeoff.

The comparison among the unfolded variants further verifies the contribution of each proposed component. Compared with the conventional deep-unfolded PGA model with J=10, introducing a trainable gradient-balancing factor improves the balance between the communication-rate gradient and the sensing-error gradient in the digital update. Incorporating the manifold-based analog update further makes the analog update more consistent with the geometry of the constant-modulus constraint.The simulation results have proved the effectiveness of these two mechanisms. The complete state-driven adaptive deep-unfolded model combines these two mechanisms and further adjusts the effective step sizes and gradient-balancing factor according to the current optimization state. Therefore, it reaches a higher joint objective and a stable high-performance region faster than the other unfolded baselines under the same number of outer iterations.

Table 1 provides a quantitative summary of the final performance and runtime of different methods. The reported values are obtained by evaluating each test sample individually and then averaging the results over the whole test set. The proposed state-driven adaptive deep-unfolded model achieves the lowest final sensing error and the highest final joint objective among all compared methods. Compared with UPGA-J10, it reduces the final sensing error by approximately 57.5% and improves the final joint objective by approximately 154.9%. Compared with UPGA-J20, it further reduces the sensing error by approximately 23.5% and improves the final joint objective by approximately 13.3%, while reducing the runtime by approximately 32.0%. This indicates that increasing the number of inner iterations alone is less efficient than the proposed state-driven adaptive design.

The proposed model also outperforms MADMM-RCG in terms of both final communication rate and final joint objective. Although the proposed model requires a slightly longer runtime than some lightweight unfolded baselines, its runtime remains moderate and is significantly lower than that of UPGA-J20. Therefore, the proposed model achieves a better performance–runtime tradeoff rather than relying on excessive computational cost.

### 5.3. Empirical Convergence Analysis

To further evaluate the empirical convergence behavior of the proposed method, we record the number of unfolded layers required to satisfy a practical convergence criterion. Based on the previously defined absolute variation of the joint objective, we further define the relative variation as

(32) $$\rho_{\left(i\right)}=\frac{\left|\Delta J_{\left(i\right)}\right|}{\left|J_{\left(i-1\right)}\right|+\epsilon },$$

where ϵ is a small positive constant introduced to avoid numerical instability.

The convergence layer $i_{\mathrm{conv}}$ is defined as the first unfolded layer at which the relative variation of the joint objective remains below a predefined threshold $\epsilon_{c}$ for *p* consecutive unfolded layers. In this paper, we set p=3. This criterion is used to characterize practical numerical stability rather than to claim theoretical convergence to a stationary point.

In Table 2, “Avg. convergence layer” denotes the average number of layers required for convergence, while “Std.” denotes the standard deviation of the required convergence layers.

As shown in Table 2, the proposed method achieves the highest convergence ratio among all compared iterative algorithms. All test samples satisfy the practical convergence criterion, while UPGA-J10, UPGA-J20, and MADMM-RCG achieve convergence ratios of 39%, 96%, and 87%, respectively. Compared with UPGA-J20, the proposed method reduces the average convergence layer from 94.46 to 62.21 while improving the convergence ratio from 96% to 100%. Although MADMM-RCG has a smaller average convergence iteration, its lower convergence ratio and larger standard deviation indicate less stable convergence behavior across test samples. These results show that the proposed method achieves more reliable empirical convergence than the baseline iterative algorithms.

Table 3 reports the empirical convergence behavior of the proposed method under different convergence thresholds over 100 independent test samples. Under the 1% criterion, all test samples reach practical convergence, with an average convergence layer of 62.21 and a standard deviation of 17.66. When a looser threshold of 5% is adopted, the proposed method reaches practical convergence within 10.53 layers on average, indicating rapid early-stage stabilization of the unfolded inference process. As the threshold becomes stricter, the required convergence layer generally increases, which is expected because a smaller threshold imposes a more stringent stability requirement. Even under the stringent 0.5% criterion, all test samples still satisfy the practical convergence condition within the maximum unfolded depth. For the very strict 0.2% criterion, 95% of the test samples reach practical convergence, with an average convergence layer of 105.81. These results demonstrate that the proposed method exhibits stable empirical convergence behavior across different convergence thresholds.

### 5.4. Effect of the Number of Sensing Targets on the Joint Objective

**Figure 7:**
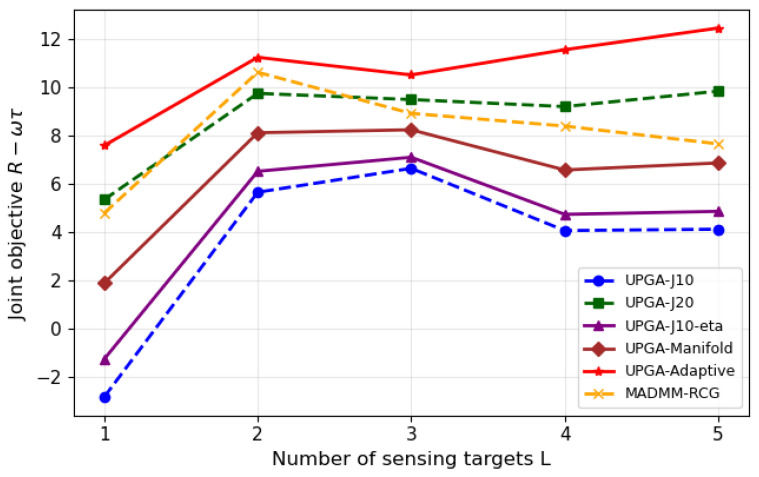
Effect of the Number of Sensing Targets on the Joint Objective.

Figure 7 shows the joint objective comparison under different numbers of sensing targets, where *L* varies from 1 to 5. This experiment evaluates the robustness of different methods under varying sensing-scene complexities.

The proposed method consistently achieves the highest joint objective across all target settings. The increasement of *L* makes the communication-sensing tradeoff harder to optimize. The advantage of the proposed method comes from its state-driven adaptive control mechanism. The state features allow the controller to adjust the analog step size, digital step size, and gradient-balancing factor according to the current optimization condition. In particular, the adaptive gradient-balancing factor changes the relative contribution of the rate-gradient and sensing-gradient terms, thereby adjusting the effective update direction when the sensing task becomes more complex.

This demonstrates that the proposed method is not tailored to a single sensing setting, but has better robustness to variations in sensing-scene complexity.

### 5.5. Joint Objective Under Different SNR Conditions

**Figure 8:**
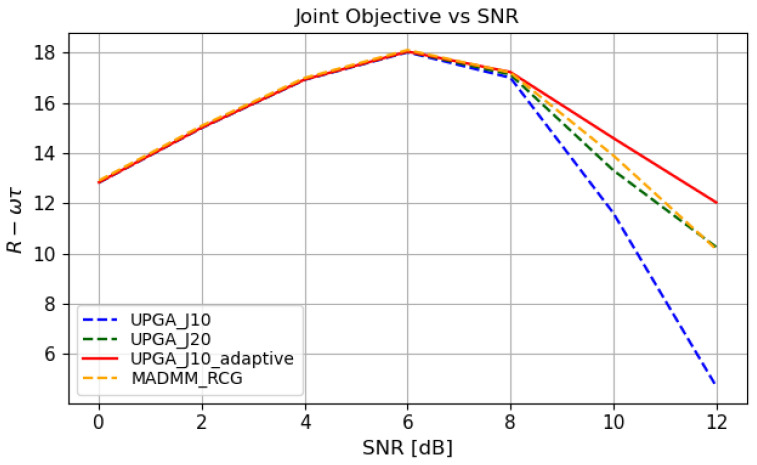
Joint objective under different SNR conditions.

Figure 8 compares the joint objective R−wτ achieved by different methods under different SNR conditions. As the SNR increases, the joint objective first increases and then decreases after reaching its peak around 6dB. In the low- and medium-SNR regions, the performance gaps among different methods are relatively small. This is because the system performance is still largely noise-limited in these regions, so the benefit brought by different precoder update strategies is not fully reflected in the joint objective.

After around 6dB, the joint objective decreases because the sensing mismatch term τ increases faster than the communication-rate gain. Under the fixed tradeoff weight *w*, this reduces the overall value of R−wτ.

Compared with the baseline methods, the proposed state-driven adaptive deep-unfolded model shows a slower degradation of the joint objective in the high-SNR region. This indicates that the proposed model provides a more stable communication–sensing tradeoff under varying SNR conditions. Such robustness benefits from the state-driven controller, which adaptively adjusts the effective step sizes and the gradient-balancing factor according to the current optimization state.

*Remark on active jamming:* The SNR-based evaluation mainly reflects passive noise-level variations. In practical MIMO-JCAS systems, active jamming and adversarial interference may also degrade communication and sensing performance. A CGAN-based fusion CNN framework is used for few-shot jamming signal classification [26]. The classified jamming type can be regarded as additional state information for describing the external interference environment. Therefore, in future jamming-aware extensions, jamming classifier outputs, together with interference-plus-noise power or received covariance features, can be incorporated into the state vector of the proposed controller to support interference-aware update control.

### 5.6. Evolution of the Scaling Factor $\alpha_{\eta }^{\left(i\right)}$

**Figure 9:**
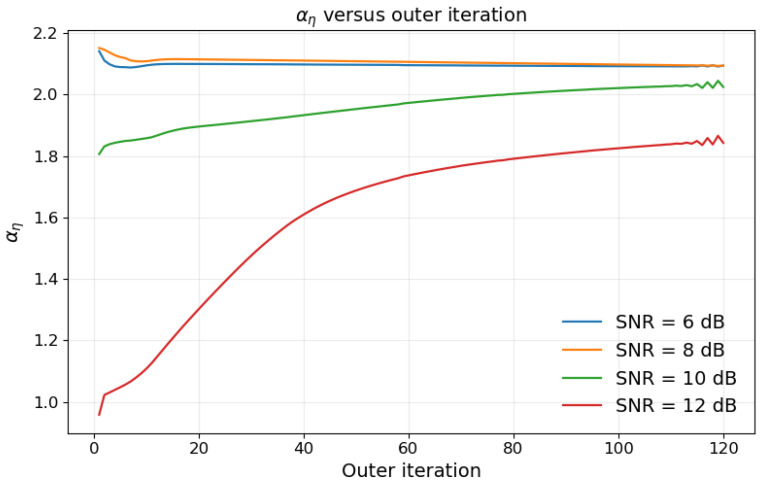
Evolution of the scaling factor $\alpha_{\eta }^{\left(i\right)}$ under different SNR conditions.

This paper further examines the gradient variations under different signal-to-noise ratio (SNR) conditions. It is observed that, as the SNR increases, the scale of variation in $\nabla_{W^{*}}\tau$ gradually becomes larger. To compensate for this gradient variation, the evolution of $\alpha_{\eta }^{\left(i\right)}$ in the iterations should exhibit a trend opposite to that of $\nabla_{W^{*}}\tau$, which is particularly evident under high-SNR conditions. By jointly considering Figure 2 and Figure 9, it can be seen that the proposed method is able to adaptively correct the imbalance between the communication gradient and the sensing gradient on the digital side, thereby improving the robustness and joint optimization performance of the model under different SNR conditions.

### 5.7. Effect of the Tradeoff Parameter w

**Figure 10:**
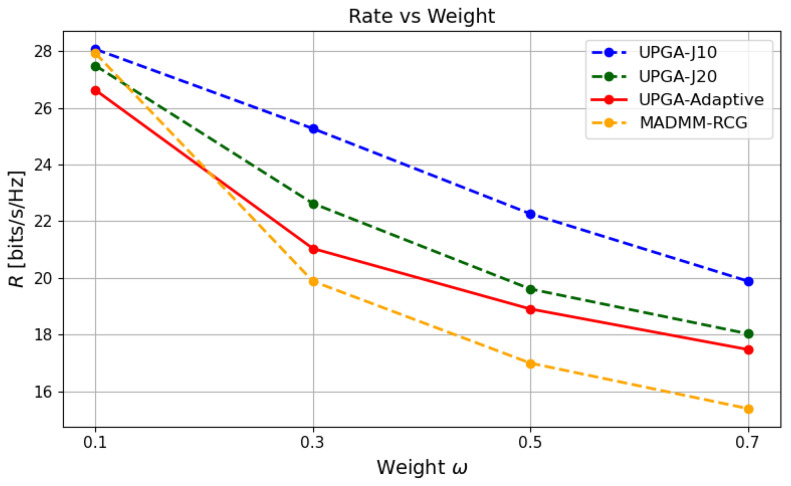
Rate versus *w*.

**Figure 11:**
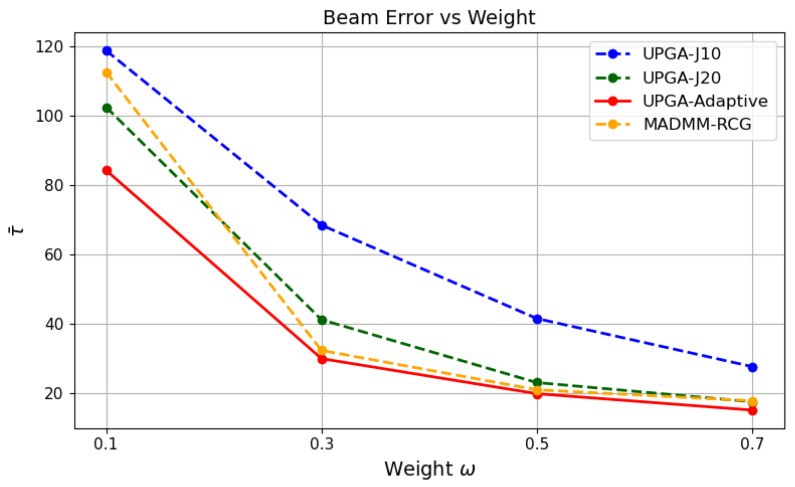
Beam error versus *w*.

**Figure 12:**
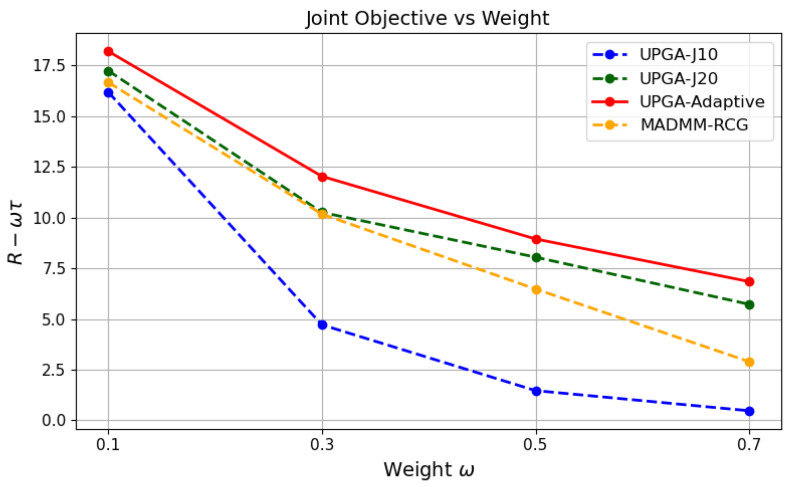
Joint objective versus the tradeoff parameter *w*.

Figure 10, Figure 11 and Figure 12 jointly evaluate the performance of different methods under different tradeoff weights *w*. As *w* increases, the optimization places more emphasis on sensing-error reduction. As a result, the sensing error generally decreases, while the communication rate decreases accordingly. This confirms that *w* controls the operating point between communication and sensing performance.

Figure 12 further shows that the proposed state-driven adaptive deep-unfolded model consistently achieves the highest joint objective across different values of *w*. This indicates good generalization to different communication–sensing preferences, rather than effectiveness only under one fixed tradeoff setting. Such generalization benefits from the state-driven controller. Different values of *w* change the relative importance of communication-rate maximization and sensing-error reduction, which requires different update behaviors during optimization. By using the current optimization state to adjust the step sizes and gradient-balancing factor, the proposed model can better match these changing communication–sensing preferences and maintain a favorable joint objective across different values of *w*.

### 5.8. Performance Under Different Channel Models

To further evaluate whether the proposed state-driven unfolded optimizer is overly specialized to the original channel distribution, we additionally test its performance under different channel models. In addition to the channel used in the main simulations, Rayleigh fading and Rician fading channels are further considered.

For the Rayleigh fading channel, each channel coefficient is independently generated from a circularly symmetric complex Gaussian distribution, i.e., $H_{k,n}∼\mathrm{CN}\left(0,1\right)$. For the Rician fading channel, both a deterministic LoS component and a scattered NLoS component are considered, and the channel is modeled as

$$h_{k}=\sqrt{\frac{\kappa }{\kappa +1}}h_{\mathrm{LoS},k}+\sqrt{\frac{1}{\kappa +1}}h_{\mathrm{NLoS},k},$$

where $h_{\mathrm{LoS},k}=\sqrt{N_{t}}a_{\mathrm{ULA}}\left(\phi_{k}\right)$ and $h_{\mathrm{NLoS},k}∼\mathrm{CN}\left(0,I_{N_{t}}\right)$. The Rician factor is set to κ=0 dB in the simulations.

To ensure a fair comparison across different channel models, all generated channel datasets are normalized to the same average per-element channel power.

**Figure 13:**
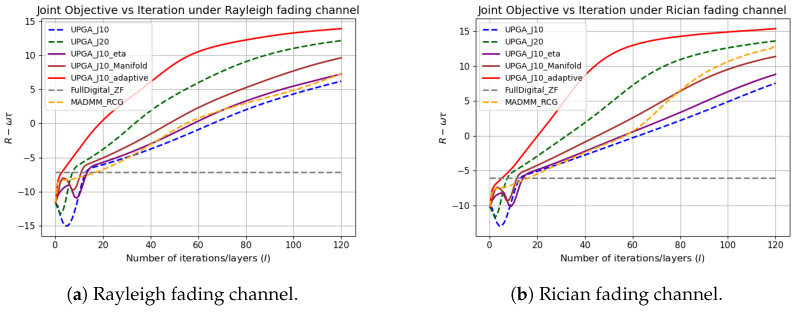
Generalization evaluation under different channel models.

Figure 13 compares the performance of different methods under the Rayleigh fading channel and the Rician fading channel. These two channel models have different statistical characteristics from the original channel. It can be seen that the proposed method consistently achieves the highest final joint objective in both channel models. The performance advantage mainly comes from the state-driven adaptive update mechanism, which dynamically adjusts the update behavior according to the current optimization state, rather than relying on fixed step sizes or a fixed communication-sensing balancing strategy. The results demonstrate that the proposed method is not limited to the original channel and can remain effective under channel models with different statistical characteristics.

### 5.9. Performance Under the Non-Uniform Linear Array (NULA) Configuration

Table 4 presents the quantitative results under the NULA configuration. Compared with the default ULA setting in Table 1, the proposed model maintains almost the same joint performance when the antenna arrangement is changed. Specifically, the final joint objective of the proposed model is 13.46 under the ULA setting and 13.40 under the NULA setting, showing only a slight decrease of about 0.45%. Meanwhile, the proposed model still achieves the lowest sensing error under the NULA configuration.

Although UPGA-J10 and UPGA-J10-η achieve higher communication rates under the NULA configuration, their sensing errors are much larger, resulting in significantly lower joint objectives. MADMM-RCG obtains a relatively low sensing error, but its communication rate and final joint objective are still inferior to those of the proposed model. Overall, the proposed model achieves the best joint objective under both the ULA and NULA configurations. This indicates that the proposed state-driven adaptive framework is not overly specialized to the original ULA setting and can maintain strong adaptability under the tested non-uniform antenna arrangement.

## 6. Conclusions

This paper proposes a state-driven adaptive deep-unfolded hybrid beamforming method for MIMO JCAS systems. The performance has been enhanced by three mechanisms: a manifold analog precoder update, a learnable gradient balancing factor for digital precoder optimization, and a state-driven adaptive hyperparameter control strategy. Simulation results demonstrate that the proposed approach achieves a superior communication-sensing tradeoff compared with conventional deep-unfolded PGA and benchmark schemes, which means higher objective values, better robustness under different SNR conditions, and faster convergence speed. Our future work will pay more attention to practical scenarios, including imperfect Channel State Information (CSI), partially connected HBF architectures, and wideband channels. 

## Figures and Tables

**Table 1:** Quantitative comparison of different methods under the default simulation setting.

Method	Final *R*	Final τ	Final R−wτ	Runtime (s)
UPGA-J10	26.91	72.11	5.28	5.48
UPGA-J20	23.91	40.08	11.88	10.37
UPGA-J10-η	26.48	66.54	6.52	5.71
UPGA-J10-Manifold	25.36	54.15	9.11	5.66
MADMM-RCG	19.54	31.85	9.98	5.89
Proposed model	22.66	30.67	13.46	7.05

**Table 2:** Empirical convergence behavior of different iterative algorithms when $\epsilon_{c}=1\%$.

Method	Test Samples	Convergence Ratio	Avg. Convergence Layer	Std.
UPGA-J10	100	39%	27.28	30.24
UPGA-J20	100	96%	94.46	11.48
MADMM-RCG	100	87%	26.83	39.36
Proposed-method	100	100%	62.21	17.66

**Table 3:** Empirical convergence behavior of the proposed method.

Criterion	Test Samples	Convergence Ratio	Avg. Conv. Layer	Std.
5% for 3 consecutive layers	100	100%	10.53	14.38
2% for 3 consecutive layers	100	100%	44.90	8.29
1% for 3 consecutive layers	100	100%	62.21	17.66
0.5% for 3 consecutive layers	100	100%	79.46	18.38
0.2% for 3 consecutive layers	100	95%	105.81	10.52

**Table 4:** Quantitative comparison of different methods under the NULA configuration.

Method	Final *R*	Final τ	Final R−wτ	Runtime (s)
UPGA-J10	26.93	73.16	4.99	5.58
UPGA-J20	23.81	40.30	11.72	10.46
UPGA-J10-η	26.48	67.44	6.25	5.69
UPGA-J10-Manifold	25.31	54.69	8.90	5.80
MADMM-RCG	19.66	34.31	9.40	5.92
Proposed model	22.48	30.28	13.40	7.12

## Data Availability

In this study, we analyzed a publicly available dataset released by the authors of a previous paper [23] and made available through their GitHub repository. The dataset can be found here: https://github.com/nhanng9115/Joint-Communications-and-Sensing-Hybrid-Beamforming-Design-via-Deep-Unfolding (accessed on 19 May 2026). After revision, we added new data created by ourselves. The raw data supporting the conclusions of this article will be made available by the authors on request. Therefore, the correct Data Availability Statement for our manuscript should be: Dataset available on request from the authors.
